# Using social network analysis of mixed-species groups in African savannah herbivores to assess how community structure responds to environmental change

**DOI:** 10.1098/rstb.2019.0009

**Published:** 2019-07-29

**Authors:** Kristine Meise, Daniel W. Franks, Jakob Bro-Jørgensen

**Affiliations:** 1 Mammalian Behaviour and Evolution Group, Department of Evolution, Ecology and Behaviour, Institute of Integrative Biology, University of Liverpool, Neston CH64 7TE, UK; 2 Department of Biology, University of York, York YO10 5DD, UK; 3 Department of Computer Science, University of York, York YO10 5GH, UK

**Keywords:** mixed-species groups, climate change, interspecific competition, antipredator strategies, social network analysis, ungulates

## Abstract

The dynamics of wildlife populations often depend heavily on interspecific interactions and understanding the underlying principles can be an important step in designing conservation strategies. Behavioural ecological studies can here provide useful insights into the structure and function of communities and their likely response to environmental changes. In this study of the Masai Mara herbivore community, we use a social network approach to investigate social affinities between species and how these change over the year in response to seasonal changes in ecological conditions. We find that even though social networks were correlated across different ecological conditions, for half the species dyads in the community, the strength of social affinities responded to changes in rainfall and/or the presence of migratory wildebeest. Several species consequentially adopted more or less central positions in the network depending on the ecological conditions. The findings point out interspecific social links that are likely to be attenuated or strengthened as a consequence of human-induced environmental changes and therefore call for particular attention from conservation managers. The eco-evolutionary ramifications of the perturbations of social affinities still require further study.

This article is part of the theme issue ‘Linking behaviour to dynamics of populations and communities: application of novel approaches in behavioural ecology to conservation’.

## 1. Background

Mixed-species groups (MSGs) constitute an integral part of the structure and function of many communities, and understanding the principles underlying their formation can therefore be of relevance to natural resource management. Different species can be driven to group either because of benefits from increased resource intake or because of reduced predation risk [1–3]. For example, passerine birds in Britain have been shown to benefit from increased information about foraging opportunities in MSGs [4], coral fish dilute individual predation risk in MSGs in which they benefit from interspecific social mimicry [5], and Amazonian primates in MSGs benefit from complementary predator detection abilities of the different species [6]. However, the payoffs from forming MSGs are likely to be context-dependent in responding to shifts in the environment and in the species composition of the community, and this may cause significant alterations in the structure and function of communities when the ecological conditions change.

To date, most studies of the relationship between ecological conditions and MSG formation have analysed social responses to human-induced changes in the environment (e.g. [3,7,8]). Several studies have found that when habitats are fragmented, there is a decrease in the proportion of MSGs, their size and the number of species participating, a pattern which has been attributed mainly to lower population densities (e.g. [9–14]). A particularly severe impact on MSG formation occurs when environmental changes affect the abundance of so-called nuclear species, i.e. species who play a central role for the cohesion of MSGs (e.g. [10,15–20]).

Changes in the prevalence and composition of MSGs in response to human impacts is of conservation relevance because they may be associated with the loss of natural ecosystem function [21–24]. Ideally, we would be able to predict cases for concern before unnatural changes happen [25]. For this purpose, it may be informative to investigate interspecific social responses to environmental change in undisturbed systems; however, little attention has been paid to this so far. Environmental changes are indeed part of natural ecosystem dynamics and are therefore expected to be reflected in the adaptations of species comprising a community [26], including their behavioural responses to each other. Among the few studies that have attempted to tease apart the nature of these adaptations, most results indicate a shift in community structures in response to changes in resource availability (e.g. [27–30]).

Uncovering the natural variation in social patterns may indicate which affiliations are likely to become more common and which are likely to disappear when given conditions within the natural range are experienced more or less often than previously [25,31]. Also, where entirely novel conditions outside the natural range are expected, the social patterns most likely to emerge may be hinted at by extrapolation based on correlations between social affinities and environmental variables within the natural range. Such changes in social constellations may have both ecological and evolutionary consequences [32,33]. In some cases, the effect on population dynamics may be only limited, but in other cases the stability of a community may be dependent on the very occurrence of natural seasonal changes [34]. Over a longer time-frame, altered social conditions are moreover expected to alter selective pressures on species and hence affect their adaptations.

The savannah herbivore community of the Serengeti-Mara region in East Africa offers a well-suited opportunity to investigate how environmental changes affect patterns in social affinities in a natural system. Not only is the system species-rich and well-known for its ubiquitous MSGs [35–37], it also undergoes drastic seasonal changes in climate as well as the presence/absence of migrants, notably wildebeest (*Connochaetes taurinus*) [38]. Focusing on the dozen most common herbivores in the system, we here use a social network approach to tease apart how environmental changes affect the propensity of individual species to form MSGs, the social affinities within specific species-dyads, and the overall centrality of individual species in the network structure.

## 2. Methods

### (a) Study system

**Figure 1.**
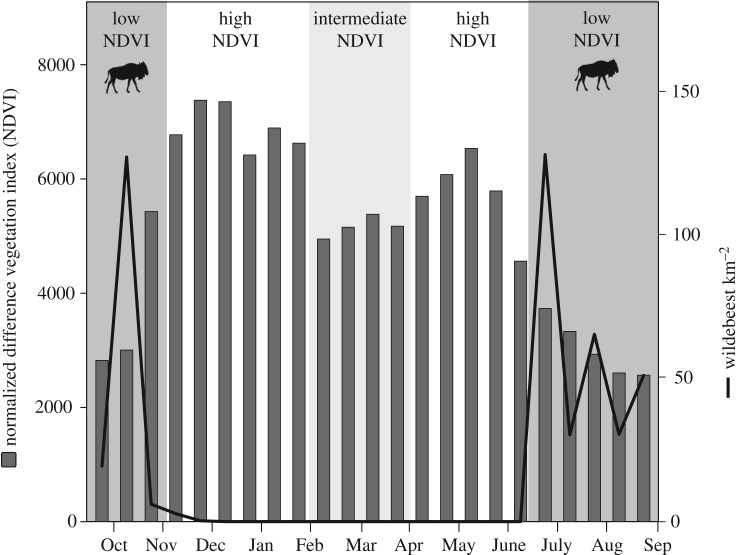
Annual variation in mean NDVI and density of wildebeest in the study area (October 2015–September 2016).

The data was collected between September 2015 and September 2016 in the Masai Mara National Reserve, south-western Kenya (1°30′ S, 35°10′ E). The ecosystem is dominated by open savannah grassland, and the year is divided into two wet seasons (typically November–January and March–May) and a short and a long dry season (typically January–March and June–October, respectively) [39]. The productivity of the grasslands is well-captured by a positive correlation with the satellite-derived Normalized Difference Vegetation Index (NDVI) [40,41]. Seasonal change in the system is furthermore characterized by the presence of the mass migration of especially wildebeest during the long dry season when the Masai Mara is favoured to the adjoining Serengeti National Park, Tanzania, owing to its generally higher rainfall [38]. For this study, we subdivided the year into three ecological conditions based on differences in mean NDVI and the presence/absence of the wildebeest migration: (i) low NDVI conditions (2500–5000; mean: 3670) during which migratory wildebeest were present, corresponding to the long dry season (September–November 2015, June–September 2016), (ii) intermediate NDVI conditions (5000–5500, mean: 5155) without wildebeest, corresponding to the short dry season (February–April 2016), and (iii) high NDVI conditions (5500–7500; mean: 6627) without wildebeest, corresponding to the two wet seasons (November 2015–February 2016; April–June 2016; figure 1).

### (b) Data collection

Over the year, we conducted a total of 66 species counts focusing on the 11 most common large herbivore species present throughout the year: Thomson gazelle (*Gazella thomsonii*, ‘Tho’), Grant gazelle (*Gazella granti*, ‘Gra’), impala (*Aepyceros melampus*, ‘Imp’), common warthog (*Phacochoerus aethiopicus*, ‘War’), ostrich (*Struthio camelus*, ‘Ost’), topi (*Damaliscus lunatus*, ‘Top’), hartebeest (*Alcelaphus buselaphus*, ‘Har’), plains zebra (*Equus quagga*, ‘Zeb’), African buffalo (*Syncerus caffer*, ‘Buf’), common eland (*Tragelaphus oryx*, ‘Ela’), and giraffe (*Giraffa camelopardalis*, ‘Gir’) [42]. In addition, we counted wildebeest, which were present during the long dry season only. The counts, which were spaced approximately 16 days apart to match the interval between successive MODIS NDVI datasets (MOD13A1, 500 × 500 m; [43]), took place on three study plains, covering a total area of 57 km^2^. We recorded the location and composition (i.e. species identity and number) of all social units using a GPS recorder (Garmin, Oregon 600) while following pre-defined tracks in a Landcruiser 4 × 4. Groups were defined by inter-individual distances less than 100 m [44], a criterion that generally distinguished them from looser aggregations. Distances were estimated by eye and confirmed using a laser rangefinder (Bushnell Scout DX 1000 ARC) whenever necessary. Migrating wildebeest alternate between aggregated travelling phases and more dispersed sedentary phases [45], and under the assumption that social affinities can be more reliably measured during the latter, we excluded from the data analysis super-herds including more than 2000 individuals, which typically could not be counted from a single vantage point. The number of individuals per count (mean ± s.e.) was 1108 ± 133 (27 counts) during high NDVI conditions, 1199 ± 180 (12 counts) during intermediate NDVI conditions, and 3717 ± 1290 (27 counts) in total during low NDVI conditions (2259 ± 323 if excluding all super-herds, and 1322 ± 165 if excluding all super-herds and all wildebeest). The number of social units per count (mean ± s.e.) was 39.7 ± 2.5 solitary individuals, 62.4 ± 4.9 single-species groups and 31.5 ± 2.6 MSGs, while the number of individuals per group (mean ± s.e.) was 9.9 ± 0.7 in single-species groups and 29.8 ± 1.7 in MSGs.

### (c) Data analysis

#### (i) Social affinity indices

We quantified the social affinity between species using a social affinity index which controls for the relative abundance of species in the ecosystem, thereby making the index comparable among species:

$$W_{\mathrm{AB}}=\left(\Sigma_{i=1}^{g}\frac{N_{i_{A}}\;*\;N_{i_{B}}}{N_{i}-1}\right)\;*\;\frac{N_{\mathrm{tot}}-1}{N_{A}\;*\;N_{B}},$$

where *g* is the number of groups in which both species A and species B are present, *N_i_* is the number of individuals in group *i*, and *N*_A_, *N*_B_ and *N*_tot_ are the total numbers of individuals of species A, of species B, respectively of all species, in the community. The expression denotes the average proportion of a social unit, experienced by an individual of species A, that consists of species B relative to the proportion of individuals of species B in the community (note the subtraction of 1 discounts for the fact that an individual of species A will by necessity group with one of its own species, namely itself, which thus does not indicate social affinity for conspecifics). The resulting index is symmetrical for any two species. We calculated social affinity indices separately for each of the three ecological conditions defined above, and identified dyads which were more or less likely to associate than expected if associations occurred at random (for calculation of *p*-values, see below). For analyses of changes in social affinities between ecological conditions, we standardized the social affinity indices to control for differences in the overall propensity of each species to form MSGs under the three ecological conditions. For this, we divided the absolute social affinity index by the sum of the species' affinity indices within the given ecological condition.

#### (ii) Hypothetical framework

**Figure 2.**
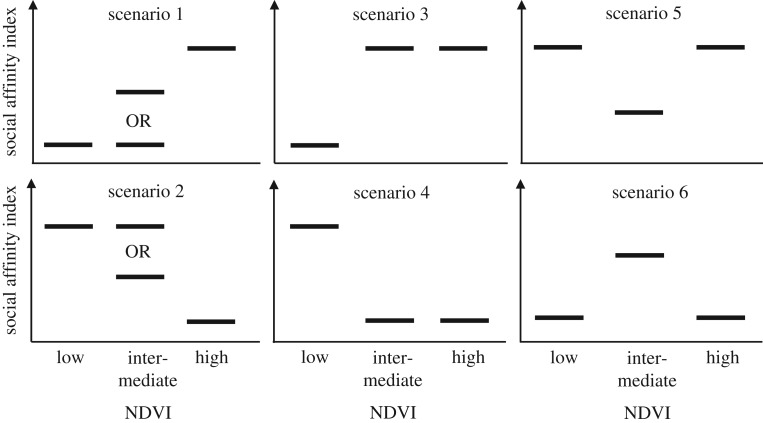
Alternative scenarios for changes in social affinity according to ecological conditions. In scenarios 1–4, social affinity changes monotonously with NDVI; however, whereas a response to NDVI is the most parsimonious explanation for the change in social affinity in scenarios 1 and 2, in scenarios 3 and 4, social affinity differs only under low NDVI conditions, and as this time is also characterized by the presence of wildebeest, a response to the wildebeest migration offers an equally parsimonious explanation in this case. In scenarios 5 and 6, the change in social affinity from intermediate to high NDVI conditions indicates either a positive (scenario 5) or negative (scenario 6) response to NDVI, and in light of that, the opposite direction of the response from low to intermediate NDVI conditions is most parsimoniously explained by an effect of the presence of wildebeest.

Changes in the standardized social affinity index between ecological conditions was used to assess the impact of rainfall and/or the presence of migratory wildebeest on the strength of social affinities between species. A hypothetical framework was derived based on the most parsimonious explanations for six possible scenarios for how the strength of the affinity index may change between ecological conditions (figure 2) as follows.

*Scenario 1:* if affinity increases from low to intermediate NDVI conditions, and again from intermediate to high NDVI conditions, rainfall is generally suggested to promote social affinity; if affinity increases from low and intermediate NDVI conditions to high NDVI conditions, but with no difference between the former, heavy rain is suggested to promote social affinity.
*Scenario 2:* if affinity decreases from low to intermediate NDVI conditions, and again from intermediate to high NDVI conditions, rainfall is generally suggested to reduce social affinity; if affinity decreases from low and intermediate NDVI conditions to high NDVI conditions, but with no difference between the former, heavy rain is suggested to reduce social affinity.
*Scenario 3:* if affinity increases from low NDVI conditions, when wildebeest are present, to intermediate and high NDVI conditions, but with no difference between the latter, the presence of wildebeest or very low rainfall is suggested to reduce social affinity.
*Scenario 4:* if affinity decreases from low NDVI conditions, when wildebeest are present, to intermediate and high NDVI conditions, but with no difference between the latter, the presence of wildebeest or very low rainfall is suggested to promote social affinity.
*Scenario 5:* if affinity is lowest under intermediate NDVI conditions, both the presence of wildebeest and high rainfall are suggested to promote social affinity.
*Scenario 6:* if affinity is highest under intermediate NDVI conditions, both the presence of wildebeest and high rainfall are suggested to reduce social affinity.

#### (iii) Statistical analysis of dyadic relations

Differences in the proportion of individuals found in a given social unit type (i.e. solitary, single- or mixed-species group) in each count was compared across the three ecological conditions for each species using Kruskal–Wallis tests.

Owing to the non-independence of dyadic association data [46,47], we used a permutation procedure to test (i) the significance of the observed social affinity of particular dyads compared to random values for each ecological condition, and (ii) the significance of changes in the standardized social affinity index between ecological conditions. The procedure randomized the group membership within ecological conditions while keeping constant the seasonal abundance of each species, the distribution of the number of conspecifics within social units, as well as the number of groups and the distribution of the number of species per group. We compared the observed value to the distribution of values obtained when running 5000 randomizations simulating that species associated randomly (following [48]). Social relations are henceforth referred to as ‘preference’ and ‘avoidance’ if the social affinity index is significantly higher, respectively lower, than expected by chance; this terminology accommodates the range of spatial drivers that may influence the propensity of species to form social associations, including shared diet and habitat preferences. Owing to differences in species abundance, *p*-values for the two species in a dyad differed slightly depending on the species in the dyad for which it was calculated; to reflect the strongest affinity, we report the lower value. Wildebeest were excluded from the analyses used to identify changes in the social relations between species present the full year; however, in order to identify the preferred social partners of the wildebeest, we ran a separate analysis of social affinity indices for the low NDVI conditions in which we included the wildebeest.

#### (iv) Network metrics describing community social structure

Focusing on the overall social network, we used Mantel tests [49,50] to test for overall Pearson's rank correlations in social affinities between species dyads across ecological conditions (*vegan* package [51]; 9999 permutations); this was done for both standardized and absolute measures of social affinities (i.e. with and without control for changes in the overall strength of social ties). In addition, we calculated the weighted degree (i.e. the sum a species' social affinity indices) as a measure of the centrality of a species within the community (*igraph* package [52]). We tested for significant differences in weighted degree between ecological conditions by comparing observed values against the distribution of values generated randomly as described above (two-tailed).

All analyses were performed in R v. 3.5.1 [53] with differences considered significant at *p* < 0.05.

## 3. Results

### (a) Group formations in relation to ecological conditions

**Figure 3.**
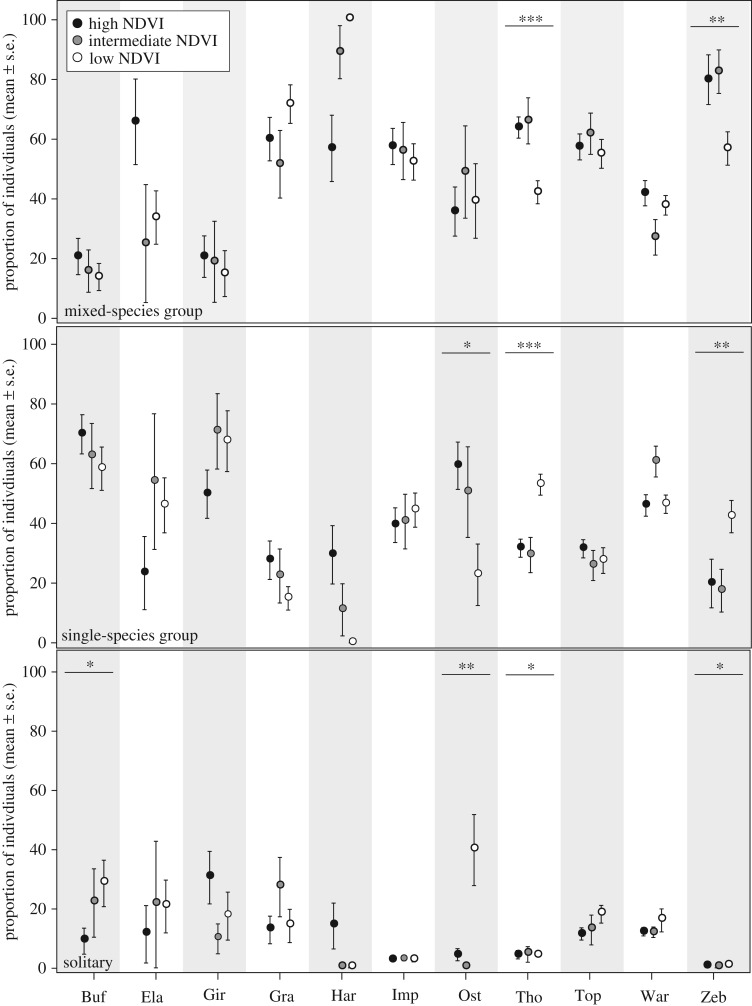
Distribution of individuals between social units according to ecological conditions (see ‘Methods’ for species abbreviations). Significant difference in the proportion of individuals in a given social unit type across ecological conditions is indicated by asterisks (*p < 0.05, **p < 0.01, ***p < 0.001).

The effect of ecological conditions on the proportion of individuals found as solitary, or in single- and mixed-species groups respectively, were modest for most species (figure 3). Two exceptions were zebra and Thomson gazelles which under low NDVI conditions were less likely to be found in MSGs and more likely to be found in single-species groups in particular. The primary explanation is likely to be the presence of large groups of migratory individuals of these two species during the long dry season, and that these experience relatively low antipredator benefits and high resource competition costs from joining heterospecifics. The ostrich was less likely to associate with conspecifics and more likely to be solitary under low NDVI conditions, which may be related to breeding and reduced intraspecific resource competition during wet conditions [54]. Similarly, buffaloes were increasingly likely to be solitary as NDVI decreased, which again may be explained by increased intraspecific resource competition as the biomass of grass decreases. It is noteworthy that no significant increases were detected in the proportions of individuals found in MSGs during low NDVI conditions, although wildebeest were only present at this time and were found in as many as 26% of all the MSGs observed (i.e. 249 of 952 MSGs).

### (b) Seasonal variation in social affinity within species dyads

Concentrating only on the species present throughout the year (i.e. excluding the wildebeest), seasonal changes in the standardized social affinity index were detected in 26 of the 55 (47%) species dyads (table 1). For nine dyads (16%), social affinity increased with increasing NDVI (scenario 1; figure 2). The eland and zebra showed a mutual preference for each other as social partners under all ecological conditions, with their affinity increasing consistently with NDVI. The affinity between warthog and topi also increased consistently with NDVI, whereas for the remaining seven dyads, affinity increased significantly under high NDVI conditions only. Eight of the dyads conforming to scenario 1 included the eland (five dyads), impala (two dyads) and/or the warthog (three dyads), and the increased social affinity may generally be explained by dietary switches in these species which allow them to join species in open habitat as conditions get wetter: the eland and impala are mixed-feeders that increasingly switch from browsing in thickets to grazing on open plains [55,56] and the warthog, which ventures further from thick vegetation, also spend more time on open plains [35]. None of the dyads demonstrated a simple increase in social affinity with decreasing NDVI as described by scenario 2. Three dyads (5%) showed reduced social affinity during low NDVI when wildebeest were present (scenario 3), again possibly because differences in feeding niches, or in this case also water dependency, lead to segregation under dry conditions: the impala increasingly switches to browsing whereas the Thomson gazelle remains predominantly a grazer, the Grant's gazelle is water-independent whereas the topi is not, and the warthog is also significantly less water-dependent than the buffalo [57,58]. These species may also conceivably differ in their tolerance of wildebeest. Another three dyads (5%) showed increased social affinity during low NDVI conditions (scenario 4). These dyads were generally composed of species that were less dependent on green grass blades, such as the gazelles, the ostrich and the warthog [58], and the changes may thus largely be explained by more arid-adapted species grouping together. For the eland and the buffalo (2% of dyads), social affinity was promoted during the presence of wildebeest as well as by high NDVI (scenario 5). This may be explained by evasion of wildebeest during low NDVI conditions, and high benefits from grouping during high NDVI conditions when the eland switches to grazing and the two species come to share, not only predators, but also the requirement for substantial quantities of grass consequential to their large body sizes [59]. Finally, for 10 dyads (18%), social affinity was reduced during the presence of wildebeest as well during high NDVI (scenario 6). Nine of these dyads included at least one of the three species that showed a preference for grouping with wildebeest, i.e. zebra (*p* < 0.001), Thomson gazelle (*p* = 0.005) and topi (*p* = 0.018); hence, the lower social affinity within these dyads during the low NDVI conditions may reflect substitution of social partners by wildebeest when these are present. The reason for the decreased social affinity during high NDVI conditions is less clear but may be partly related to divergent sward preferences when variation in grass height becomes more pronounced.

**Table 1.** Social affinity within species dyads according to ecological conditions. (For diagrammatic representation of scenarios, see figure 2. Note that only dyads showing significant changes between ecological conditions are shown. + and − denote preference, respectively avoidance. ↗ and ↘ denote increase, respectively decrease, in standardized affinity indices between ecological conditions. n.s.: not significant.)

	dyad	valence of social relation			change in social affinity		
		low NDVI	intermediate NDVI	high NDVI	low versus intermediate NDVI	low versus high NDVI	intermediate versus high NDVI
scenario 1	eland/zebra	+, *p* = 0.039	+, *p* = 0.007	+, *p* = 0.001	↗, *p* = 0.041	↗, *p* < 0.001	↗, *p* < 0.001
	eland/Thomson gazelle	n.s.	n.s.	+, *p* = 0.035	n.s.	↗, *p* = 0.022	↗, *p* = 0.015
	eland/warthog	n.s.	n.s.	+, *p* = 0.007	n.s.	↗, *p* = 0.012	↗, *p* = 0.001
	eland/impala	n.s.	n.s.	+, *p* = 0.024	n.s.	↗, *p* = 0.016	↗, *p* = 0.016
	impala/hartebeest	n.s.	n.s.	+, *p* = 0.030	n.s.	↗, *p* = 0.023	↗, *p* = 0.023
	warthog/hartebeest	n.s.	n.s.	+, *p* = 0.018	n.s.	↗, *p* = 0.023	↗, *p* = 0.019
	eland/Grant gazelle	n.s.	n.s.	n.s.	n.s.	↗, *p* = 0.029	↗, *p* = 0.036
	Thomson gazelle/zebra	+, *p* = 0.022	n.s.	+, *p* = 0.002	n.s.	↗, *p* < 0.001	↗, *p* = 0.005
	warthog/topi	n.s.	n.s.	+, *p* = 0.001	↗, *p* = 0.027	n.s.	↗, *p* = 0.001
scenario 3	impala/Thomson gazelle	+, *p* < 0.001	+, *p* < 0.001	+, *p* < 0.001	↗, *p* = 0.007	↗, *p* = 0.022	n.s.
	warthog/buffalo^a^	−, *p* = 0.038	n.s.	n.s.	n.s.	↗, *p* = 0.019	n.s.
	topi/Grant gazelle^b^	n.s.	+, *p* = 0.018	+, *p* = 0.042	↗, *p* = 0.019	n.s.	n.s.
scenario 4	Grant gazelle/ostrich	+, *p* = 0.019	n.s.	n.s.	↘, *p* = 0.004	↘, *p* = 0.004	n.s.
	warthog/Thomson gazelle	+, *p* = 0.006	n.s.	n.s.	↘, *p* = 0.019	↘, *p* = 0.015	n.s.
	zebra/Grant gazelle	+, *p* < 0.001	n.s.	n.s.	↘, *p* = 0.022	↘, *p* = 0.021	n.s.
scenario 5	eland/buffalo	+, *p* = 0.010	n.s.	+, *p* = 0.025	↘, *p* = 0.007	↘, *p* = 0.015	↗, *p* = 0.013
scenario 6	Thomson gazelle/hartebeest	n.s.	+, *p* = 0.004	n.s.	↗, *p* < 0.001	n.s.	↘, *p* < 0.001
	Thomson gazelle/topi	+, *p* < 0.001	+, *p* < 0.001	+, *p* < 0.001	↗, *p* < 0.001	↗, *p* < 0.001	↘, *p* = 0.020
	topi/hartebeest	n.s.	+, *p* = 0.027	n.s.	↗, *p* = 0.010	n.s.	↘, *p* = 0.009
	topi/impala	n.s.	+, *p* < 0.001	+, *p* < 0.001	↗, *p* < 0.001	↗, *p* < 0.001	↘, *p* < 0.001
	topi/ostrich	n.s.	+, *p* = 0.016	n.s.	↗, *p* = 0.012	n.s.	↘, *p* = 0.024
	topi/zebra	+, *p* < 0.001	+, *p* = 0.002	+, *p* = 0.025	↗, *p* = 0.020	n.s.	↘, *p* = 0.012
	zebra/giraffe	n.s.	+, *p* = 0.012	n.s.	↗, *p* = 0.017	n.s.	↘, *p* = 0.014
	zebra/hartebeest	+, *p* = 0.028	+, *p* = 0.038	n.s.	↗, *p* = 0.016	n.s.	↘, *p* = 0.046
	zebra/ostrich	n.s.	+, *p* = 0.032	n.s.	↗, *p* = 0.020	n.s.	↘, *p* = 0.025
	Grant gazelle/hartebeest	n.s.	+, *p* = 0.029	n.s.	↗, *p* = 0.009	↘, *p* = 0.039	↘, *p* = 0.006

^a^Categorized as scenario 3 owing to lack of significant change between intermediate and high NDVI conditions and low affinity during low NDVI conditions only.

^b^Categorized as scenario 3 owing to lack of significant change between intermediate and high NDVI conditions, during both of which affinity was high.

### (c) Seasonal variation in social structure of the community network

**Figure 4.**
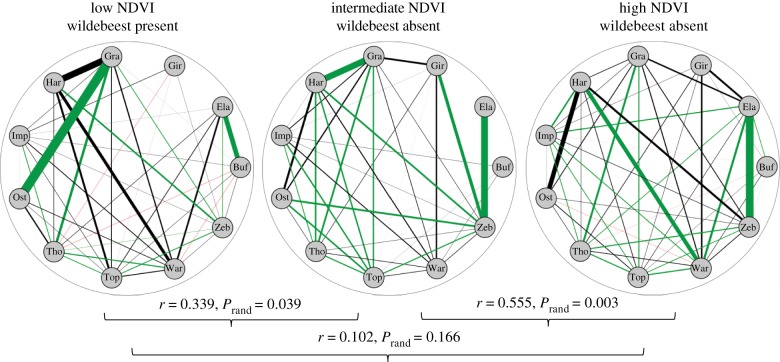
The social affinity network of the savannah herbivores under different ecological conditions. Green and red indicate preferred, respectively avoided, associations. Significance of correlations is based on Mantel tests. Only species present in the community throughout the year are included; see ‘Methods’ for species abbreviations and details on statistical analysis.

**Figure 5.**
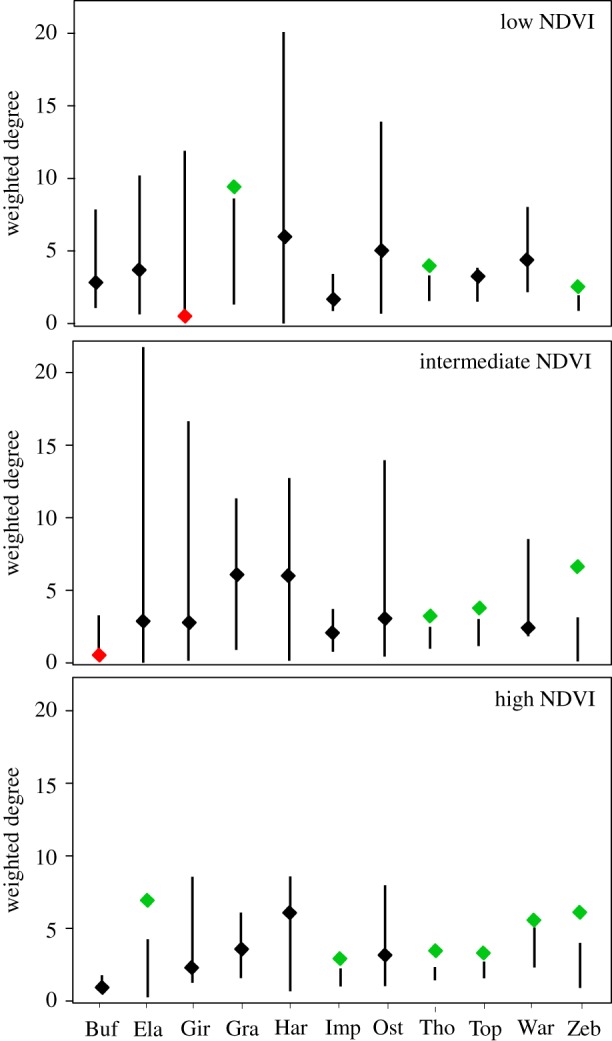
Weighted degree (a measure of centrality) of the species in the social affinity networks (figure 4). Black bars indicate the 95% range of the expected values if species associated randomly. Diamonds show the observed values with green and red indicating species significantly more, respectively less, central than expected by chance.

The standardized affinity indices were correlated across the community between all ecological conditions (Mantel test, low versus intermediate NDVI conditions: *r* = 0.5776, *p* < 0.001; low versus high NDVI conditions: *r* = 0.564, *p* < 0.001; intermediate versus high NDVI conditions: *r* = 0.677, *p* < 0.001), as were the absolute social affinity indices except for the comparison between the low and high NDVI conditions (figure 4). These correlations suggest a degree of stability of social relations between species across ecological conditions, with the most significant changes occurring between the low and high NDVI conditions. However, the weighted degree for several species differed significantly between seasons, indicating a change in their centrality in the network (figure 5). Eland, impala and warthog occupied significantly more central positions under high NDVI than under low or intermediate NDVI conditions (difference in weighted degree, eland: low versus high NDVI conditions: −3.25, *p* = 0.017, intermediate versus high NDVI conditions: −3.87, *p* = 0.011; impala: low versus high NDVI conditions: −1.12, *p* = 0.011, intermediate versus high NDVI conditions: −0.61, *p* = 0.050; warthog: intermediate versus high NDVI conditions: −2.86, *p* = 0.009; figure 5). This result agrees well with the positive effect of rainfall on social affinity in dyads including these species (see above). The three species which showed a preference for associating with the wildebeest, i.e. zebra, Thomson gazelle, and topi, generally occupied central positions in the network of species present throughout the year (figure 5), although the zebra significantly less so during low NDVI conditions (difference in weighted degree, low versus intermediate NDVI conditions: −4.63, *p* < 0.001; low versus high NDVI conditions: −3.80, *p* = 0.006), a pattern which may be explained by the influx of migratory individuals that more often form single-species groups (figure 3) or group with the wildebeest. Finally, the Grant's gazelle was more central under low NDVI conditions, which is consistent with the increased affinity between arid-adapted species at this time (see above).

## 4. Discussion

Our study reveals that the social affinities of all the study species from the African savannah herbivore community were affected by changes in ecological conditions. Thus, social affinity increased with rainfall in several dyads including mixed feeders who switched to grazing on open plains during wetter conditions. For other dyads, which included preferred associates of the wildebeest, substitution by wildebeest as social partners offers an explanation for a decrease in social affinities for resident species during the long dry season. Yet, other more arid-adapted, species strengthened their social ties during dry conditions. As a result, the centrality of several species in the network depended on the ecological conditions, even if we only detected significant seasonal changes in the proportion of individuals in MSGs in a minority of species. These findings demonstrate the sensitivity of the social structure in the community to environmental change (see also [36]). From a conservation perspective, this context-dependence of interspecific social relations is of concern because the Serengeti-Mara ecosystem is confronted with drastic anthropogenic changes to the environment which, by affecting the social structure of the herbivore community, may have adverse consequences for the stability and functionality of the ecosystem. In the following, we discuss the possible consequences of two of the most important threats that the ecosystem is facing, namely habitat fragmentation and climate change.

Ongoing habitat fragmentation owing to road construction [60,61] and, in particular, fencing [62,63] is having a devastating impact on the connectivity in the Serengeti-Mara ecosystem at present and poses an imminent threat to the persistence of the wildebeest migrations. If the influx of migratory wildebeest to the Masai Mara during low NDVI conditions is reduced, the social constellations that become more common may include those that we suggest may currently be attenuated by the presence of wildebeest (scenario 3), especially those ties otherwise promoted by dry conditions (scenario 6). Disfavoured social links, on the other hand, may include that between the buffalo and eland which we suggest may partly be driven by both species evading the wildebeest (scenario 5). A limitation of our study, however, is that the coincidence of low NDVI conditions with the presence of wildebeest prevents any firm conclusions about the drivers of changes in social relations during the long dry season to be reached.

Over a longer time-frame, human-induced climate change is predicted to have an intensifying impact on environmental conditions [64], with the consensus prediction for East Africa being that rainfall will increase ([65]; however, see [66]). According to our analyses, this may lead to a closer integration of some mixed-feeders into the interspecific social network (scenario 1), whereas social ties between more arid-adapted species (scenario 4) and various other species (scenario 6) may become weaker. A factor likely to contribute to such a pattern is that the migration of wildebeest is expected to remain longer in the Serengeti if rainfall increases, because the move to the relatively wet Masai Mara is driven by dry conditions when the short-grass plains in Serengeti become void of free water [38].

Our study thus identifies likely changes in social relations between species owing to human activities, and these will conflict with the goal of conservation in so far as they interfere with natural ecological and evolutionary processes. An important next step in forecasting eco-evolutionary changes is to quantify the effect of MSGs on vital rates, as this is essential for the prediction of population dynamic consequences. Also by making assumptions about rates of evolutionary change, likely evolutionary consequences can be modelled. Incorporating dynamics of MSG formation in ecological studies can moreover shed light on wider ecosystem-level processes; for example, the effect of social information use on foraging behaviour in fishes has been shown to affect nutrient cycling [67,68]. In our study system, vegetation structure is known to respond to grazing pressure, with dramatic results when ecological tipping points are reached [69], and changes in the patterns of MSG formation may here have important consequences which are not immediately obvious. Modifications of interspecific contact rates can also affect the persistence of transmissible diseases [70,71,72], and in savannah herbivores, the number of helminthic parasite species shared is known to depend on the propensity of host species to form MSGs [73].

In conclusion, this study illustrates the value of taking a community-wide approach in behavioural ecological studies aiming to inform biodiversity conservation, and not focusing just on single species of conservation concern. Species do not exist in isolation and, because loss of preferred social partners can lower survival, either by increasing predation risk or reducing foraging efficiency, the impact of environmental changes can only be fully understood if analysed within a multi-species framework [74,75]. We believe that social network analysis here provides a useful framework with rich scope for further development to better predict population performance, and ultimately evolution, of individual species within communities undergoing perturbations. To inform practical conservation, integration of social network analysis with complementary disciplines relevant to concrete issues shows great promise for identifying the critical features in need of protection to achieve conservation goals. Notably, when it comes to making spatially explicit recommendations, the application of a social network approach in landscape ecology is likely to yield valuable insights [76]. For such analyses involving interspecific social associations, we believe that a particular advance in our study is the multi-species social affinity index which we developed to account for the proportion of individuals of different species forming MSGs in relation to their proportion in the community at large. Most studies on MSGs so far have based their analysis simply on co-occurrences of different species, whereby precious information is lost.

## Data accessibility

Data are available from the Environmental Information Data Centre (EIDC) at: https://doi.org/10.5285/cc0794f0-748a-42aa-b491-2f9b65c771a6.
